# Case of Diabetes Mellitus Successfully Treated by the Nitric Acid

**Published:** 1800-09

**Authors:** Nicholas Chavasse

**Affiliations:** Surgeon in Walfall, and Member of the Company of Surgeons in London


*Case of Diabetes Mellitus successfully treated by the Nitric Acid; communicated to* Dr. GILBY *by* NICHOLAS CHAVASSE, Surgeon in Walsall, and Member of the Company of Surgeons in London.

### CASE.

Thomas Pitt, aged 64, applied to me about ten weeks since, with an inveterate Diabetes. He had been a hard drinker for more than twenty years, and had laboured under a profuse discharge of water for thirteen months. When he first consulted me, he was making nine quarts of sweet urine in twenty-four hours: His pulse was weak and frequent; his thirst excessive; appetite very bad, and appearance cachectic. He was directed to live upon animal food, to abstain from vegetables, sugar, and all fermenting liquors; but at any time, when unusually faint, to drink a few glasses of Sherry. He took volatile and fixed alkali alternately in the day, and opium at bed-time. As the latter medicine produced costiveness, he had recourse to the flowers of sulphur to remove that inconvenience. He persisted in following these rules for twelve days; but as oedematous swelling of the lower extremities came on, accompanied with increased debility, hectic fever, and urinous secretion, I thought it prudent to abandon this plan. He was now directed to take every two hours, two ounces of a mixture, composed of a drachm of nitric acid, a quart of water, and one ounce of the syrup of ginger; to relinquish the use of his opium; to eat any kind of food his stomach would prefer; to drink porter at his meals, and four glasses of Port wine after dinner. At the expiration of a week, he called on me again; I found his general health much improved, and the quantity of urine evacuated was diminished to seven quarts. Fearing the acid might irritate the bowels, the use of it was reduced to every four hours; and he continued taking that dose for twelve days longer, when I found him convalescent, and making only five quarts of water in the day and night. Suspecting that this favourable change might be the result of a change of diet, I directed him to eat and drink as he had done the last nineteen days, to discontinue all medicines, and to call on me again in a week. I conjectured erroneously, for five days had scarcely elapsed, when the anasarcous symptoms, and increased secretion of urine both returne.d He then regularly gularly perſiſted in taking the nitric acid every four hours for a month; when the efficacy of the medicine was as conſpicuous as before. He now enjoys better health than he has done for years, and his flow of urine is as moderate as it ever had been. Now we are upon the ſubject of nitric acid, permit me to ſay, I cannot, with truth, unite my evidence to thoſe who have given ſuch flattering accounts of its efficacy in the cure of inveterate ſyphilis; I have tried it, and fairly tried it, in a great number of caſes of lues, in different ſtages of the diſeaſe; and the reſult of all my experience convinces me, that it has no ſpecific powers in curing that diſeaſe. When the conſtitution has been broken down by violent pain, and the exceſſive uſe of mercury, then I know it will relieve local ſymptoms, merely by improving the general health: But here, the mercury muſt be laid aſide, and the ſame good effects may be gained by the vitriolic acid, the bark, or both united; or by ſteel medicines; all of which we know have a tendency to invigorate the digeſtive organs. I do not find, by comparative trials, that the nitric acid poſſeſſes any ſuperiority over the medicines I have enumerated.

I ſhall be happy to find, that the experience of other practitioners will enable them to ſpeak as highly of the nitric acid in the cure of Diabetes as I, with truth, have done in this ſolitary caſe.

